# Disulfiram alleviates acute lung injury and related intestinal mucosal barrier impairment by targeting GSDMD-dependent pyroptosis

**DOI:** 10.1186/s12950-022-00313-y

**Published:** 2022-10-20

**Authors:** Jiping Zhao, Hong Wang, Jintao zhang, Fuwei Ou, Junfei Wang, Tian Liu, Jinxiang Wu

**Affiliations:** 1 Department of Pulmonary and Critical Care Medicine, Cheeloo College of Medicine, Qilu Hospital, Shandong University, Jinan, China; 2 Department of Ophthalmology, Cheeloo College of Medicine, Qilu Hospital, Shandong University, Jinan, China; 3 Department of Respiratory, Cheeloo College of Medicine, Shandong Qianfoshan Hospital, Shandong University, Jinan, China; 4 Yanzhou Branch of Affiliated Hospital of Jining Medical University, Jining, China

**Keywords:** Acute lung injury, Intestinal mucosal barrier impairment, Pyroptosis, GSDMD, Disulfiram, Inflammation

## Abstract

**Background:**

Pyroptosis was implicated in acute lung injury (ALI). Disulfiram is reported as an effective pyroptosis inhibitor by inhibiting gasdermin D(GSDMD). However, the function of pyroptosis executor GSDMD and treatment of disulfiramon on ALI, especially whether it was involved in ALI-associated intestinal mucosal barrier impairment remains unclear. This study aims to explore the role of pyroptosis and disulfiram’ treatment on ALI and related intestinal mucosal barrier impairment.

**Methods:**

First, we established lipopolysaccharide (LPS)-induced ALI models in wild-type and *Gsdmd* knockout (*Gsdmd*^−/−^), to detect the effect of pyroptosis on ALI-related intestinal mucosal barrier impairment. Furthermore, we used wild-type mice treated with disulfiram to investigate the treatment of disulfiram on ALI and related intestinal mucosal barrier impairment.

**Results:**

The data showed that GSDMD-mediated pyroptosis was activated in both lung and intestinal mucosa tissues in LPS-induced ALI, and deficiency of *Gsdmd* ameliorated LPS-induced ALI and related intestinal mucosal barrier damage. We also disclosed that disulfiram inhibited the pyroptosis level, and alleviated ALI and related intestinal mucosal barrier impairment induced by LPS.

**Conclusion:**

These findings suggested the role of GSDMD-mediated pyroptosis and the potential application treatment of disulfiram in ALI and related intestinal mucosal barrier damage.

**Supplementary Information:**

The online version contains supplementary material available at 10.1186/s12950-022-00313-y.

## Background

Acute lung injury (ALI), a common complication of sepsis, is an inflammatory life-threatening disease, due to excessive activation of immune cells and overproduction of inflammatory cytokines, which ultimately results in the dysfunction or damage of multiple organs, including kidneys, heart, and gut [1]. ALI can influence the composition of the gut micro biota, drive gut dysbiosis, and then cause the damage of intestinal mucosal barrier [2]. Its underlying mechanisms may involve tissue damage, different infectious pathogenic microorganisms, their toxins [3], and uncontrolled activation of the lung inflammatory cells response [4, 5]. Pyroptosis also play an important role in the pathogenesis of ALI and related organs impairment.

Pyroptosis is an inflammatory form of programmed cell death mediated by gasdermin D(GSDMD), which is an important member of the gasdermin family, and initiated downstream of inflammasome assembly in activated innate immune cells [6, 7]. Pyroptsis changes the intracellular structure to kill intracellular bacteria and destroy the replication environment to resist pathogens, which exhibits a role of immune defense [8, 9]. It has emerged as a powerful defense mechanism of the host against microbial pathogens, and drives detrimental auto-inflammation and sepsis.[10] Many researches showed that pyroptosis is associated with acute lung injury, which involved in caspase-1, caspase-5, ASC, and NLRP1 [11]. Cleavage of GSDMD induces release of the active membrane pore-forming GSDMD peptide, which leads to lytic death of cells swelling [12, 13] by promoting passive secretion of IL-1β, IL-18 and alarmins [14, 15]. However, the function of GSDMD in ALI and subsequent intestinal mucosal barrier remain unclear.

Disulfram is a drug to treat alcohol addiction and an inhibitor of GSDMD, it has no effect on other GSDMs. Thus, it prevents the release of inflammatory cytokines and inhibiting cell pyroptosis [16]. As our work reported [17], until now, the treatment of disulfram in the ALI and related organ dysfunction is unreported.

According to the findings and data, we first employ both *Gsdmd*-knockout (*Gsdmd*-KO) mice and wild-type (WT) to detect the function and mechanism of GSDMD-dependent pyroptosis on the LPS-induced ALI, and secondary intestinal mucosal barrier damage. At the same time, we first explore the therapeutic effect of pyroptosis inhibitor disulfiram in the LPS-induced ALI and subsequent intestinal mucosal barrier damage.

## Methods

### Animals

C57BL/6 mice used in these experiments were obtained from the animal center of Shandong University. *Gsdmd*^*−/−*^ mice were provided by Dr. Feng Shao (Investigator and Deputy Director for Academic Affairs, NIBS, Beijing, China) and fed in the animal facility at the Shandong University. Mice were cared for under specific pathogen-free (SPF) conditions and allowed free access to normal laboratory diet. The study complied with the approval of the Institutional Animal Care and Use committee of Shandong University.

### Reagents

LPS (Escherichia coli O55:B5) was obtained from Sigma-Aldrich (St. Louis, MO, USA). Disulfiram (DSF) was purchased from Delta F Corporation (Xian, China). Antibodies (Abs) against mouse IL-1β, IL-18, N-GSDMD, ZO-1 and Occludin used for Western blot analysis and immunofluorescence are from BioLegend (San Diego, CA).

### Animal experimental protocol

*Gsdmd*^*−/−*^ mice and C57BL/6 mice were randomly divided into the following groups (n = 10/group): WT control, WT + LPS, *Gsdmd*^*−/−*^ control and *Gsdmd*^*−/−*^ +LPS. The mice were anesthetized by inhalation with 2% isoflurane, and administered with intraperitoneal (i.p.) injection of LPS (10 mg/kg, 50 µL) (for WT + LPS, *Gsdmd*^*−/−*^+LPS groups) or PBS (for WT control, *Gsdmd*^*−/−*^ control group). The mice were sacrificed, and a midline laparotomy was performed at 24 h after LPS injection with or without the additional treatments.

To explore the effects of DSF on ALI-induced pyroptosis in the lung and intestinal epithelium, C57BL/6 mice were randomly divided into the following groups (n = 10/group): WT control, WT + LPS, WT + DSF (50 mg/kg), WT + DSF (25 mg/kg) + LPS, WT + DSF (50 mg/kg) + LPS, WT + DSF (100 mg/kg) + LPS. The mice were anesthetized by inhalation with 2% isoflurane, and were intervened by intraperitoneally (i.p.) injection DSF (25 mg/kg, 50 mg/kg and 100 mg/kg, respectively) (for WT + DSF, WT + DSF + LPS groups) or PBS (for WT control, WT + LPS groups) 24 h prior to LPS injection. Then the mice were administered with intraperitoneal (i.p.) injection of LPS (10 mg/kg, 50 µL) (for WT + LPS, WT + DSF + LPS groups) or PBS (for WT Control, WT + DSF groups). The C57BL/6 mice were sacrificed, and a midline laparotomy was performed at 24 h after LPS injection with or without the additional treatments. Blood, lung tissue, small intestine and bronchoalveolar lavage fluids (BALF) samples of mice were obtained for histopathological, molecular and biological experiments.

### Serum and BALF collection

At the indicated time points, mice were sacrificed, and the trachea was externalized. Then 0.5–0.8 ml of ice-cold, sterile PBS was injected inside lungs and collected in clean tubes. The above procedure was repeated four times for each mouse. Total leukocytes were counted with 10 µl of BALF in a hemocytometer and the differential counts were performed on cytospin slides stained with DiffQuik reagent under light microscopy. Blood samples were also collected. The blood samples and remaining BALF were centrifuged at 450* g* for 10 min, then cell-free supernatants and serum was collected and preserved at -80℃ for cytokine analysis.

### Human samples

Twenty-three patients with ARDS secondary to Gram-negative sepsis and twenty patients without lung disease were recruited from Qilu Hospital of Shandong University, and intubation and mechanical ventilation were performed simultaneously. Blood samples from all patients were collected in sodium citrate tubes and centrifuged at 3500 rpm for 15 min, and serum was stored at -80 °C for further analysis. All studies on human subjects were approved by the Institutional Review Committee of Qilu Hospital of Shandong University.

### ELISA analysis

The production of IL-6 (cat. no. ab178013 (human), cat. no. ab46100(mouse)), IL-1β (cat. no. ab108865 (human), cat. no. ab197742(mouse)), IL-18(cat. no. ab215539(human), cat. no. ab216165(mouse)), TNF-α (cat. no. ab285327) and human GSDMD (cat. no. ab272463) were measured with ELISA kits (Abcam, Cambridge, UK) according to the manufacturers’ instructions. DAO (cat. no. YS02542B, Yaji Biological China) and D-lactic acid (cat. no. E1112821, R-Biopharm China) were also detected with ELISA kits according to the manufacturers’ instructions.

### Histological analysis of lung injury

The lung and small intestine of mice were fixed with 4% neutral buffered formalin immediately, then embedded with paraffin and cut into 5 μm sections. The sections were stained with hematoxylin and eosin (H&E) staining for histological analysis. Pathological changes evaluation of lung tissue was performed and lung injury score was calculated on the following histologic features: alveolar capillary hyperemia, alveolar wall thickness, infiltration of inflammatory cells and red blood cells into the airspace and hyaline membrane formation [18]. The lung injury score was calculated on a 0- to 4-point scale: scope of lesions < 25%, 1; 25–50%, 2; 50–75%, 3; >75%, 4, and the total score was the sum of all items mentioned above. Scoring was conducted in a blinded manner.

### Lung wet/dry ratios

Four hours after water injection, the left lung lobes were taken out and weighed immediately, then dried at 50℃ for 72 h to constant weight, and the lung weight was weighed again. The wet/dry ratio was calculated by dividing wet weight by dry weight.

### Immunofluorescence staining

IL-1β, IL-18, ZO-1 and Occludin in the lung and small intestine were identified by immunofluorescence using primary antibodies (1:300 dilution) including anti-IL-1β (cat. no. ab9722, Abcam), anti-IL-18(cat. no. ab71495, Abcam), anti-ZO-1(cat. no. ab216880, Abcam) and anti-occludin (cat. no. ab222691, Abcam). After washing, the slides were incubated with secondary secondary antibody for 1 h at room temperature in the dark. Then the lung and small intestine tissues were stained with 40, 6-diamidino-2-phenylindole (DAPI) and observed with a fluorescence microscope.

### Western blot analysis

Lung and small intestine tissue lysate were separated by electrophoresis and detected with primary antibodies as described previously [17]. The primary antibodies used were as following: Abs against IL-1β (1:1,000), IL-18 (1:1,000), N-GSDMD (cat. no. ab210070, Abcam) (1:1,000) and GAPDH (cat. no. ab9485, Abcam). The secondary horseradish peroxidase-conjugated antibodies were obtained from Applygen Technologies Inc. (Beijing, China). The original images of Western blot in our research were provided as supplemental materials (supplemental material 1).

### Statistical analyses

Study design and sample sizes were determined on the basis of our previous study, and all experiments were replicated at least three times to confirm findings. All quantitative data were presented as mean ± standard deviation (SD), and statistical analyses were performed using GraphPad Prism software version 7.0 (GraphPad Software, San Diego, CA). Student’s unpaired t test was used to evaluate the difference between two groups. Differences between multiple groups with one variable were determined using one-way ANOVA followed by post hoc Newman-Keuls Multiple Comparison Test. The Kruskal–Wallis test followed by Dunn’s Multiple Comparison Test was used for non-Gaussian Distribution. The cell number of different cell types in BALF was evaluated using two-way ANOVA test with Bonferroni correction for multiple comparisons. The value of *p < 0.05* were considered significant.

## Results

### The pyroptosis level is significantly increased with the severity of the Acute Lung Injury Patients

**Fig. 1:**
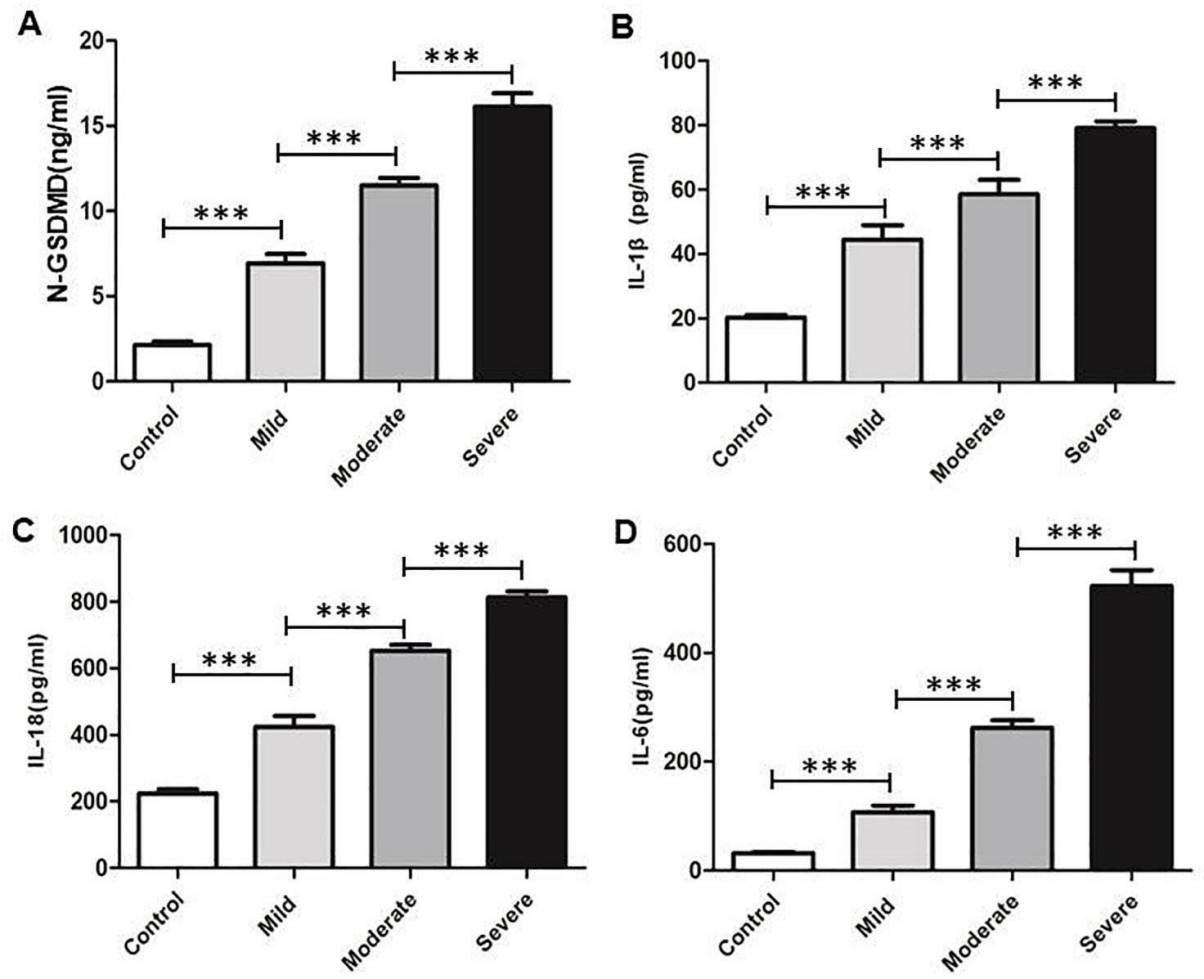
Pyroptosis level is increased with the severity in ALI patients. Detection of (**A**)N-GSDMD, (**B**)IL-1β, (**C**) IL-18 and (**D**)IL-6in serum in ALI patients were measured by ELISA. All data are median values from 3 independent experiments. ***P < 0.001, by one-way ANOVA followed by post hoc Newman-Keuls Multiple Comparison Test

Firstly, we sought to determine whether the plasmatic N-GSDMD and pyroptosis related inflammatory cytokines IL-18 and IL-1β levels differed between ALI groups and Control group. The plasmatic N-GSDMD(Fig. 1 A), IL-18 (Fig. 1B)and IL-1β (Fig. 1 C)levels were higher in the ALI (*p < 0.0001*) as compared to the Control group, and the levels were increased accompany with the severity of the ALI. Meanwhile, the infection cytokines IL-6 expression in plasma were augmented in the ALI groups (*p < 0.0001*) compared to Control group (Fig. 1D).

### LPS increases GSDMD expression and deficiency of *GSDMD* attenuates LPS-induced inflammation in acute lung injury

**Fig. 2:**
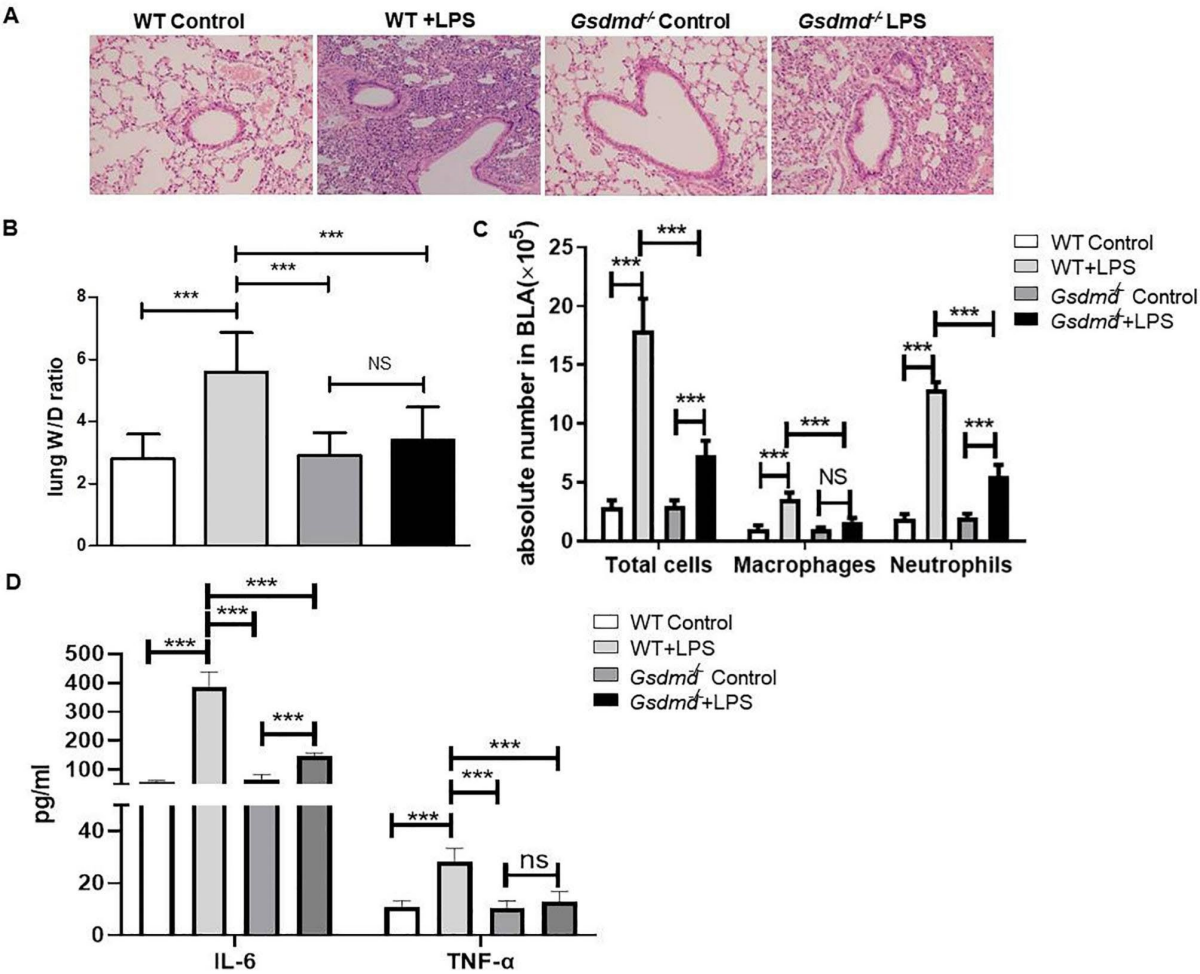
GSDMD on LPS-induced pyroptosis is required in ALI. (**A**) H&E staining of lung sections from WT and *Gsdmd*^*−/−*^ groups mice with or without LPS administration under x200 magnification (scale bar = 100 μm);(**B**) wet/dry Ratio of the lung, ***P < 0.001, by one-way ANOVA followed by post hoc Newman-Keuls Multiple Comparison Test; (**C**) Levels of the number of total cells, macrophages, and neutrophils in BALF, ***P < 0.001, by two-way ANOVA followed by post hoc Bonferroni’s Multiple Comparison Test ; (**D**) Expression of IL-6 and TNF-α in serum, ***P < 0.001, by one-way ANOVA followed by post hoc Newman-Keuls Multiple Comparison Test n = 10 for per group. All data are median values from 3 independent experiments. NS means Not significant

To determine the status of pyroptosis and effects of GSDMD on LPS-induced ALI, we established LPS-induced ALI model in mouse models. Lung tissue was histologically examined with H&E staining. Consistent with published findings, hemorrhage, pulmonary edema, alveolar wall thickening, and neutrophil infiltration were increased in the WT treated with LPS group compared with the Control group [19], and then GSDMD deficiency can attenuate LPS-induced ALI (Fig. 2 A). The lung W/D weight ratio(Fig. 2B), and number of total cells, macrophages, neutrophils in BALF(Fig. 2 C), as well as the expression of IL-6 and TNF-α (Fig. 2D) in plasma were significantly increased in the WT-LPS groups compared with the other groups,which was effectively decreased by *Gsdmd*-knockout.

### *GSDMD* knockout ameliorated acute lung injury related intestinal mucosal impairment

**Fig. 3:**
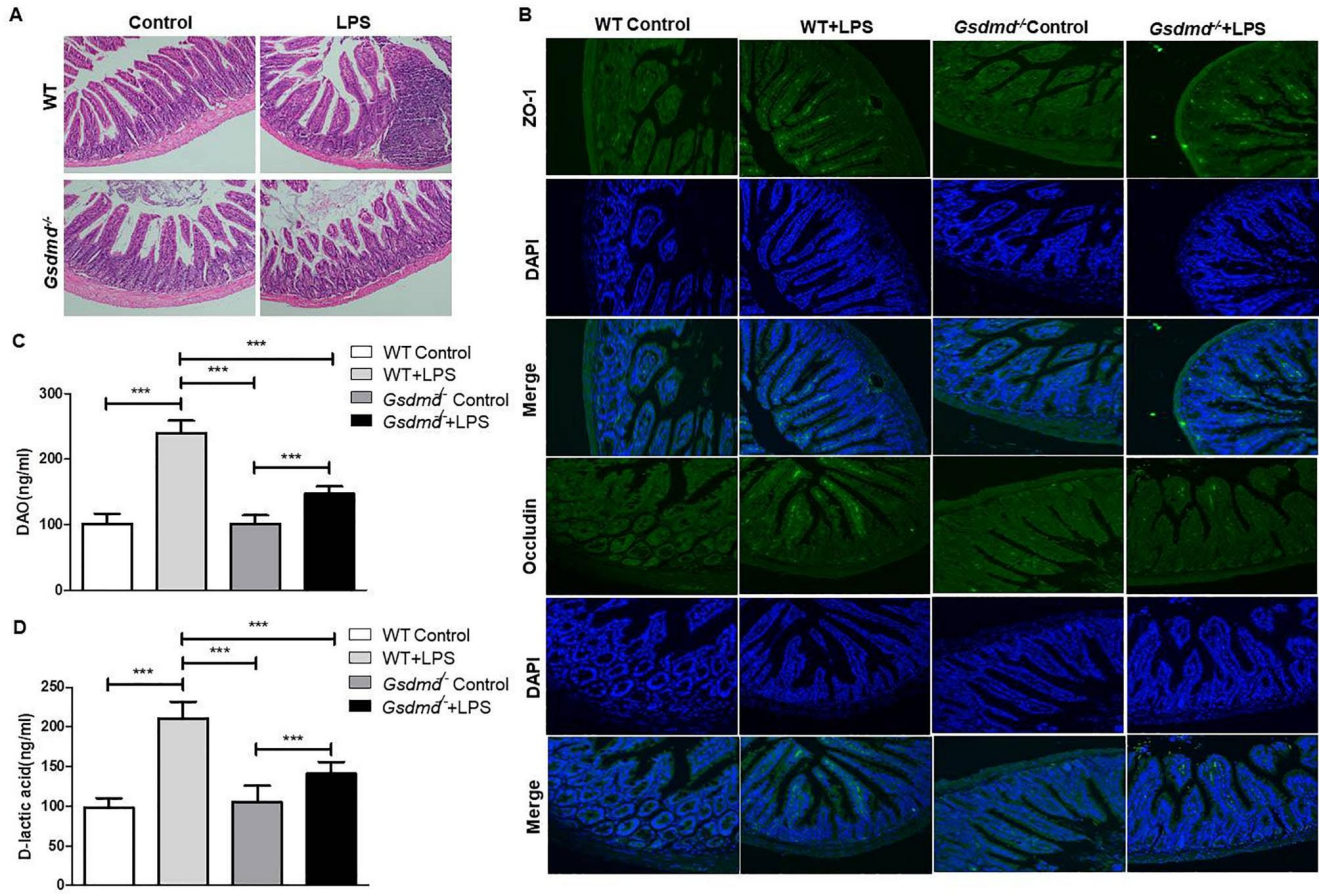
GSDMD was required for ALI-induced intestinal barrier damage. (**A**) H&E staining images of intestinal mucosa tissues from WT and *Gsdmd*^*−/−*^ groups mice with or without LPS treatment, under x200 magnification (scale bar = 100 μm); (**B**) The protein levels of ZO-1 and Occludin in intestinal mucosa tissues from indicated mice were determined by immunofluorescence histochemistry under x200 magnification (scale bar = 100 μm); The alveolar-capillary permeability by measuring(**C**) DAO and (**D**) D-lactic acid concentration. ***P < 0.001, by one-way ANOVA followed by post hoc Newman-Keuls Multiple Comparison Test

Through our research, intestinal barrier damage was observed in ALI. But it remains unclear whether GSDMD-dependent pyroptosis plays a role in ALI-induced intestinal barrier, we next investigated pyroptosis level in intestine mucosal epithelium. Intestine mucosal tissue was histologically examined with H&E staining. Intestinal damage was characterized by a reduction in villus height, villus epithelial lifting/loss, lamina propria swelling and a large amount of neutrophil infiltration into the mucosa. Intestinal mucosal impairment level was significantly increased in the WT-LPS groups compared with the other groups, which was effectively decreased by *Gsdmd*-knockout (Fig. 3 A). Consistently, the expression of ZO-1 and Occludin, two markers of intestinal mucosal barrier, significantly increased in WT-LPS groups, compared to controls, and *Gsdmd* deficiency decreased the expression of ZO-1 and Occludin (Fig. 3B). We speculated intestinal and alveolar-capillary permeability by measuring DAO and D-lactic acid in serum, we found DAO and D-lactic acid expression were significantly elevated in the WT-LPS group, and attenuated by *Gsdmd* Knockout (Fig. 3 C-D).

### *GSDMD* is required for LPS-induced pyroptosis in ALI and related intestinal mucosal impairment

**Fig. 4:**
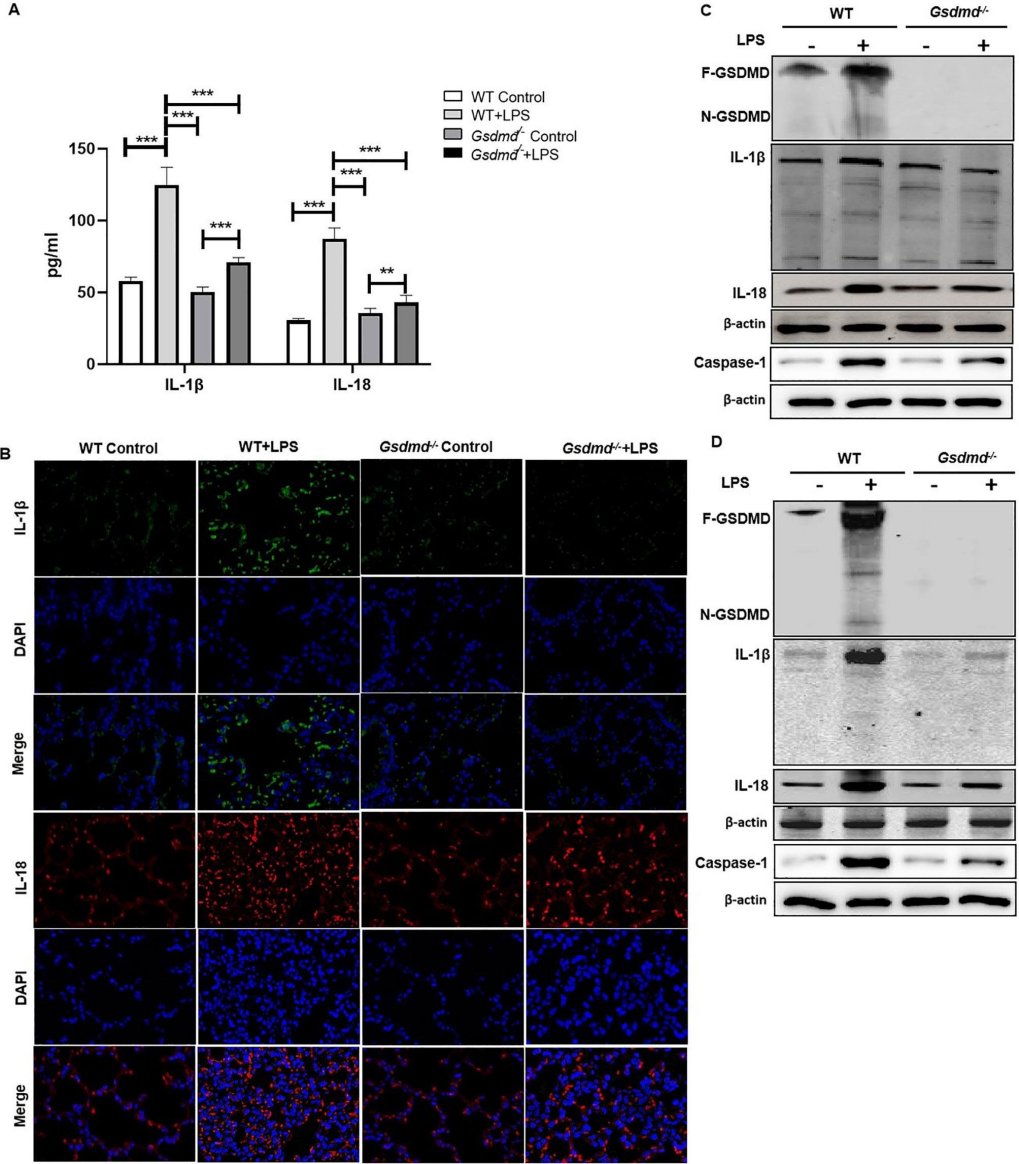
*Gsdmd* deficiency inhibited pyroptosis level in LPS-induced ALI and related intestinal barrier impairment. (**A**) Levels of pyroptosis relative inflammatory cytokines IL-18 and IL-1β in serum, **P < 0.01, ***P < 0.001, by one-way ANOVA followed by post hoc Newman-Keuls Multiple Comparison Test; (**B**)The expression of IL-18 and IL-1β were quantified by immunofluorescence histochemistry and (**C**) expressions of N-GSDMD, IL-18, IL-1β and caspase-1 were detected by Wb in lung tissue of WT and *Gsdmd*^*−/−*^ groups mice. (**D**) The expression of N-GSDMD, caspase-1, IL-18 and IL-1β were detected by Wb in intestinal mucosa tissue of WT and *Gsdmd*^*−/−*^ groups mice

To explore the effects of GSDMD on LPS-induced pyroptosis in the lung of ALI and ALI-induced intestinal mucosal dysfunction, we found that the pyroptosis inflammatory factors, IL-1β and IL-18 were increased in the serum and lung tissue of WT-LPS group, and *Gsdmd* deficiency reversed the high expression (Fig. 4 A&B). Activation of caspase-1 as the feature of cell death induced by pyroptosis, we also detect the expression of caspase-1 [20]. Meanwhile, similar result of IL-18, IL-1β, N-GSDMD and caspase-1 levels was obtained using Wb in the lung tissue (Fig. 4 C). Then, we got the similar results in intestinal epithelium, IL-18, IL-1β, caspase-1 and N-GSDMD protein was also notably increased in the WT-LPS group and was substantially inhibited by *Gsdmd* Knockout (Fig. 4D).

### Disulfiram protected mice from acute lung injury induced by LPS

To investigate the treatment effect of disulfiram on lung inflammation in ALI, we detected the severity of ALI among the *Gsdmd*^*−/−*^ groups and WT groups treat with disulfiram. Disulfiram was administered at a dose of 25, 50, or 100 mg/kg at 0 h, 12 h before LPS administration, mice were anesthetized at 24 h after LPS challenge. The lung inflammation and cytokines of TNF-α and IL-6 levels were markedly down-regulated at dose of 50 mg/kg in the WT mice.

**Fig. 5:**
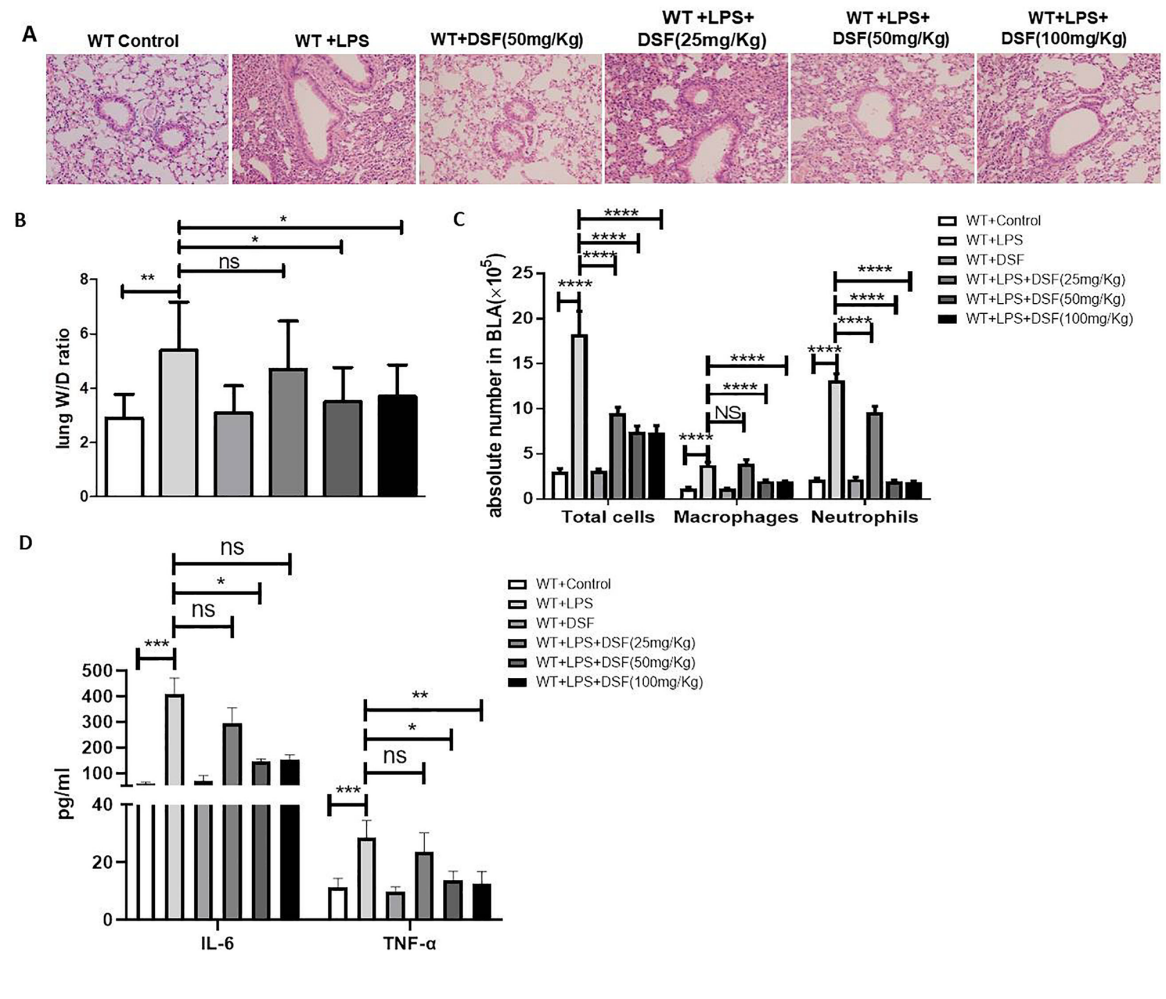
Disulfiram ameliorated lung inflammation in WT mice treated with LPS. (**A**) H&E staining of lung sections from indicated mice under x200 magnification (scale bar = 100 μm);(**B**) wet/dry Ratio of the lung, *P < 0.05, **P < 0.01, by Kruskal-Wallis test followed by Dunn’s Multiple Comparison Test; (**C**) Levels of the number of total cells, macrophages, and neutrophils in BALF, ***P < 0.001, ****P < 0.0001, by two-way ANOVA followed by post hoc Bonferroni’s Multiple Comparison Test; (**D**) Expression of TNF-α and IL-6 in serum. n = 10 for per group. *P < 0.05, **P < 0.01, ***P < 0.001, by one-way ANOVA followed by post hoc Newman-Keuls Multiple Comparison Test; NS means Not significant

We demonstrated that disulfiram could significantly alleviated LPS-induced lung inflammation in ALI (Fig. 5A). The lung W/D weight ratio (Fig. 5B), the number of total cells, macrophages, neutrophils in BALF(Fig. 5 C), and serum TNF-α and IL-6 were reduced by disulfiram treatment (Fig. 5D). The data demonstrated that disulfiram’ treatment on ALI was effective.

### Disulfiram alleviated ALI related intestinal mucosal impairment

**Fig. 6:**
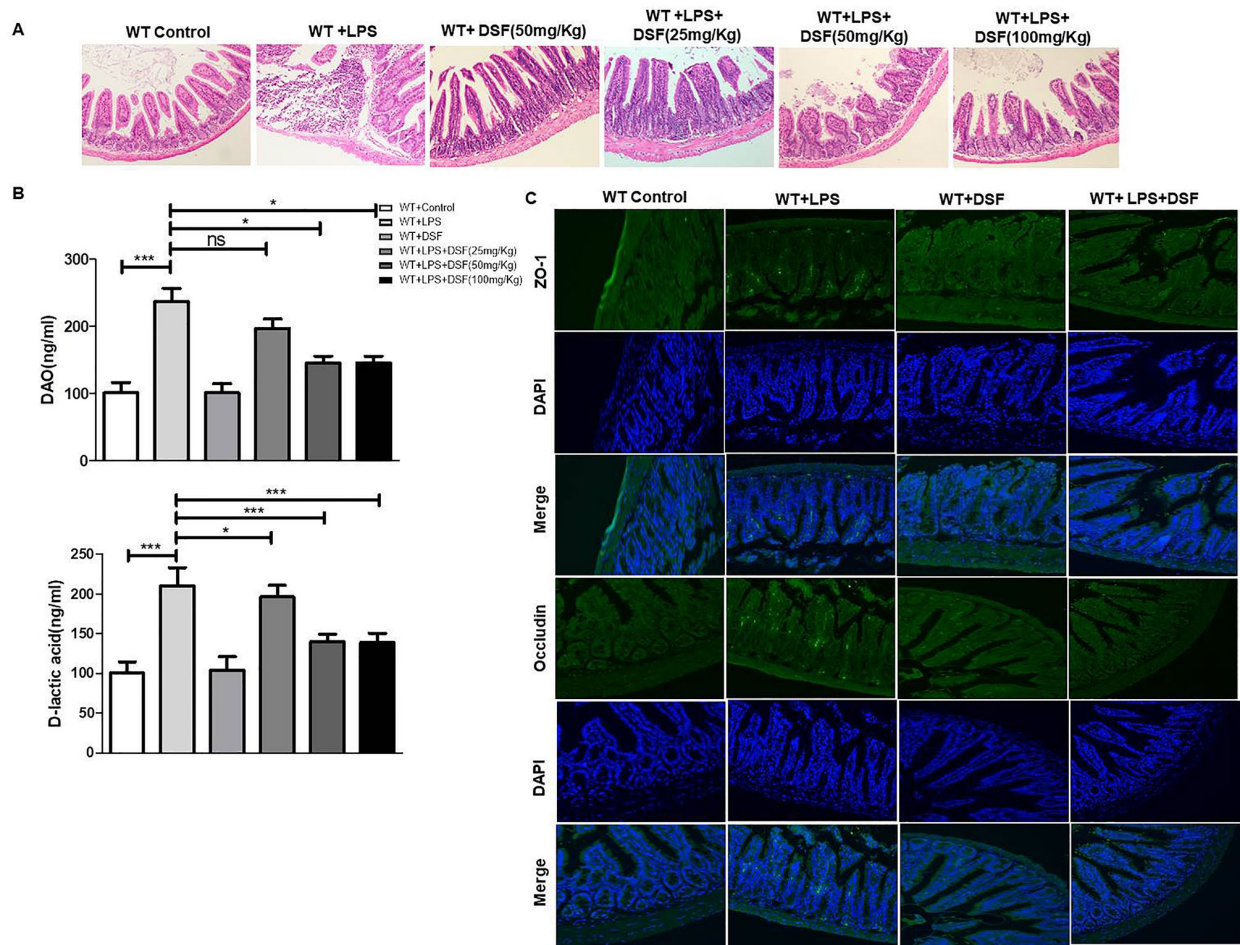
Disulfiram alleviated ALI-induced intestinal mucosal impairment. (**A**) H&E staining images of intestinal mucosa tissues from indicated mice under x200 magnification (scale bar = 100 μm); (**B**) The alveolar-capillary permeability by measuring DAO and D-lactic acid concentration, *P < 0.05, ***P < 0.001 by one-way ANOVA followed by post hoc Newman-Keuls Multiple Comparison Test; NS means Not significant. (**C**) The protein levels of ZO-1 and Occludin in intestinal mucosa tissues from indicated mice were determined by immunofluorescence histochemistry, under x200 magnification (scale bar = 100 μm)

To determine the function of disulfiram on ALI-induced intestinal mucosal dysfunction, histological analyses showed that ALI-induced intestinal inflammation was restored by disulfiram administration compared with the control group in WT mice (Fig. 6 A). Meantime, we assessed intestinal epithelial barrier permeability by examining DAO and D-lactic acid in serum, WT ALI mice exhibited higher concentrations of serum DAO and D-lactic acid than those in control mice, and disulfiram significantly decreased the expression of DAO and D-lactic acid (Fig. 6B).

Compared with controls, the expression of ZO-1 and Occludin in the intestinal mucosa of ALI mice were up-regulated, while that of ZO-1 and Occludin decreased by disulfiram (Fig. 6 C). Thus, disulfiram may play an important role on the treatment of intestinal epithelial barrier dysfunction induced by ALI.

### Disulfiram reduced the level of pyroptosis-induced by LPS-induced ALI.

**Fig. 7:**
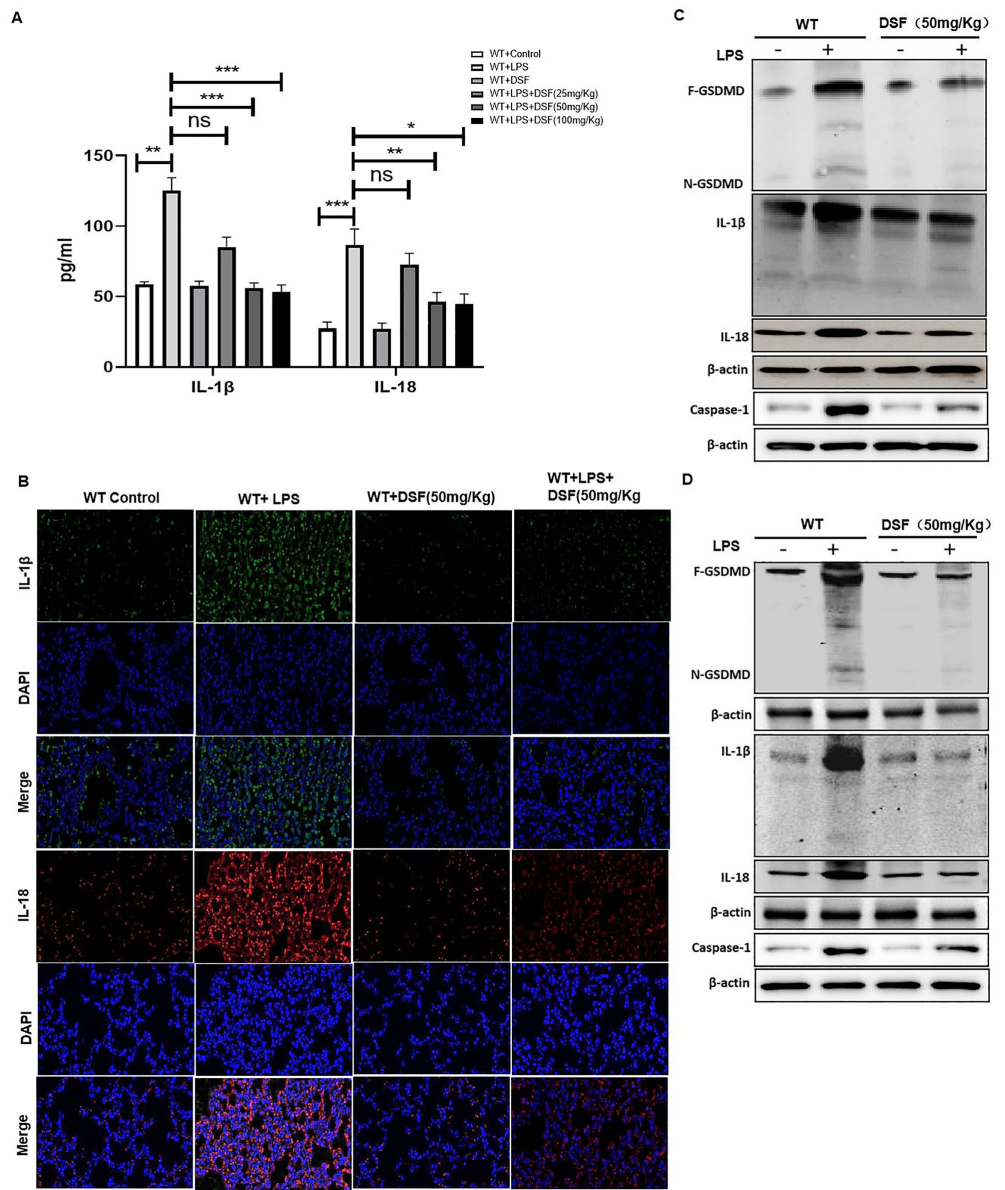
Treatment with disulfiram decreased pyroptosis level in LPS-induced ALI and related intestinal mucosa impairment. (**A**) Levels of pyroptosis relative cytokines IL-18 and IL-1β in serum by ELISA, *P < 0.05, **P < 0.001, ***P < 0.001 by one-way ANOVA followed by post hoc Newman-Keuls Multiple Comparison Test NS means Not significant; Detection of IL-18 and IL-1β in lung tissue of WT mice were quantified by (**B**) immunofluorescence histochemistry under x200 magnification (scale bar = 100 μm) and (**C**)expressions of N-GSDMD, IL-18, IL-1β and caspase-1 were explored in lung tissue of WT mice by Wb; (**D**)The protein levels of N-GSDMD, caspase-1, IL-18 and IL-1β in intestinal mucosa tissues were also detected by Wb

To explore the effects of disulfiram on ALI-induced pyroptosis in the lung and intestinal epithelium, we found that the pyroptosis inflammatory factors, IL-1β and IL-18 were increased in the serum and lung tissue of WT-LPS group, and disulfiram reversed the high expression (Fig. 7 A&B). Similar result of IL-18, IL-1β, caspase-1 and N-GSDMD in the lung tissue was obtained using Wb in the lung tissue (Fig. 7 C). Meanwhile, we tested the pyroptosis level of in ALI-induced intestinal epithelium. The expressions of IL-18, IL-1β, caspase-1 and N-GSDMD in intestine mucosal epithelium tissue were significantly increased in the LPS group, which was effectively decreased by disulfiram treatment (Fig. 7D).

## Discussion

Acute lung injury is a leading cause of death in endotoxemia-induced lung sepsis due to the wholesale destruction of the lung and related organs dysfunction [21]. Pyroptosis is a form of cell death that features plasma membrane rupture and release of proinflammatory intracellular contents. A central pathogenic feature underlying ALI is the increased pyroptosis level of lung, and subsequent exaggerated lung inflammation and injury [22]. However, their role in the pathogenesis of ALI remains unknown. Here, we tested the hypothesis that GSDMD is required for the induction of ALI. In the canonical or non-canonical pathway of pyroptosis, exogenous pathogens and endogenous damage are recognized by the intracellular sensor proteins, and then activate caspase-1/4/5/11. Activated caspases cleave GSDMD, also cleave pro-inflammatory cytokines IL-1β and IL-18, leading to maturation of IL-1β and IL-18 released from the pores on the cell membrane and amplify the local or systemic inflammation [20]. As typical pyroptosis markers, IL-18, IL-1β and N-GSDMD expressions were increased in serum accompany with the severity of the ALI patients, when compared with healthy subjects. In this study, we showed that LPS induced pyroptosis through GSDMD in ALI animal model, and *Gsdmd* deficiency could ameliorate LPS-induced ALI in the lung. Our data showed that circulating N-GSDMD level could be a biomarker for pyroptosis in ALI/ARDS patients and help stratify patients who may benefit from therapeutic interventions to contain pyroptosis.

ALI might contribute to multisystem dysfunction, septic shock, and the systemic inflammatory storm that occurs in the second phase of the ARDS infection and which is in part responsible for the disease’s mortality. In recent years, it has become more evident that the intestine can play a critical role in infectious disease, including the lung [23]. Gut disorders during ALI infection might also participate in concomitant or secondary bacterial infections, which develop in severely ill patients, such as COVID-19 [24–27]. Our research consistent with former studies that intestinal mucosal impairment could be found in LPS induced ALI. But mechanisms through which the lung could influence the gut intestinal mucosal dysfunction remains unclear, and no studies reported the involvement of GSDMD-mediated pyroptosis in the pathogenesis of ALI-induced intestinal mucosal impairment. We further confirm the crucial role of GSDMD-mediated pyroptosis in ALI-associated intestinal mucosal disfunction by using GSDMD-knock-out mice. We found *Gsdmd* deficiency could ameliorate intestinal mucosal dysfunction induced by ALI. The data suggested that pyroptosis plays a central role in the homeostasis of intestinal mucosa induced by ALI.

The discovery of GSDMD as the final common step in pyroptosis and release IL-1β/IL-18 raised a novel approach for targeted therapy of ALI-associated intestinal mucosal impairment. So, there is an urgent need to provide new insights on ALI pathogenesis and explore the possible therapeutic strategies for the disease.

Disulfiram, as a drug used to treat alcohol addiction, is a pore-formation inhibitor of GSDMD and has no effect on other GSDMs [16]. Recent reports have shown the crucial role of disulfiram in inflammatory disorders. Our team indicated that both *Gsdmd* knockout and treatment with disulfiram inhibited GSDMD-mediated pyroptosis, and alleviated the injury of the pancreas and lungs in severe acute pancreatitis models [17]. Jing Zhang et al. found that disulfiram can alleviate corneal epithelial cell damage through inhibiting the NLRP3-ASC-caspase-1-GSDMD pyroptosis pathway [28]. A study showed that DSF combined with Cu was shown to be a underlying inhibitor of the functional proteasomes by activation of NF-κB pathway, where proteasomes are involved in degradation of the inhibitor-KB molecule (IkB) in many cancers [29, 30].Other studies have indicated that disulfiram can inhibit inflammation and fibrosis in renal fibrosis rats and unilateral unilateral obstruction model by inhibiting GSDMD[31].

The mechanisms of disulfiram on the treatment of ALI are unclear. Our research found that disulfiram can inhibit the expression of GSDMD and prevents the release of related inflammatory cytokines from ALI-induced pyroptosis in lung and intestinal mucosa. Meantime, it was confirmed in our study that disulfiram not only alleviated the injury of the ALI but also protected ALI-associated intestinal mucosal impairment, which meant that treatment of disulfiram could prevent the disease from mild to severe, reduce the proportion of patients with severe conditions, and reduce mortality. It is worth looking forward to further conduct clinical trials.

Recent studies showed that macrophage-mediated pyroptosis is significant for the removal of certain intracellular bacterial and viral infections, including Legionella pneumophila and influenza [32]. Current studies suggested that alveolar macrophages also can determine the outcome and severity of disease [33]. Pyroptosis is also commonly observed in infected macrophages and dendritic cells, depending on the caspase-1 activation [34]. However, the mechanism of GSDMD in LPS-induced ALI remains elusive. The limitation of this research is that the function and molecular or immune signaling pathways of pyroptosis in ALI were needed to be further studied in-vivo in future.

## Conclusion

Taken together, we provided direct evidence showing increased pyroptosis in ALI patients and correlated to the severity. Secondly, we reported on GSDMD-mediated pyroptosis involved in the progression of ALI and associated intestinal mucosal impairment. Thirdly, inhibition of pyroptosis using disulfiram or *Gsdmd* knock out had an ameliorating effect on the inflammation of ALI and associated intestinal mucosal impairment. The data suggested that pyroptosis suppression might be applied as a possible treatment for alleviating ALI and the injury of intestinal mucosa.

## Electronic supplementary material

Below is the link to the electronic supplementary material.

Supplementary Material 1

## Data Availability

The datasets used and/or analyzed during the current study are available from the corresponding author on reasonable request.

## Abbreviations

ALI: Acute lung injury
GSDMD: Gasdermin D
LPS: Lipopolysaccharide
ASC: Apoptosis-associated speck‐like protein containing a CARD
NLRP1: Nucleotide-binding domain leucine‐rich repeat pyrin domain containing 1
IL-18: Interleukin 18
IL-1β: Interleukin 1 beta
TNFα: Tumor necrosis factor alpha.
WT: Wild-type
KO: Knock-out
DSF: Disulfiram
ZO-1: Zona occludens 1
BALF: Bronchoalveolar lavage fluid
ARDS: Acute respiratory distress syndrome
DAO: Diamineoxidase
H&E: Hematoxylin and eosin
DAPI: 6-diamidino-2-phenylindole
GAPDH: Glyceraldehyde-3-phosphate dehydrogenase
SD: Standard deviation
NLRP3: Nod-like receptor (NLR) pyrin domain-containing 3
PBS: Phosphate-buffered saline
