# Correction: Disulfiram Alleviates Acute Lung Injury and Related Intestinal Mucosal Barrier Impairment by Targeting GSDMD-Dependent Pyroptosis

**DOI:** 10.1186/s12950-024-00410-0

**Published:** 2024-09-27

**Authors:** Jiping Zhao, Hong Wang, Jintao zhang, Fuwei Ou, Junfei Wang, Tian Liu, Jinxiang Wu

**Affiliations:** 1 Department of Pulmonary and Critical Care Medicine, Cheeloo College of Medicine, Qilu Hospital, Shandong University, Jinan, China; 2 Department of Ophthalmology, Cheeloo College of Medicine, Qilu Hospital, Shandong University, Jinan, China; 3 Department of Respiratory, Cheeloo College of Medicine, Shandong Qianfoshan Hospital, Shandong University, Jinan, China; 4 Yanzhou Branch of Affiliated Hospital of Jining Medical University, Jining, China

**Correction** **:** **J Inflamm 19, 17 (2022)**

**https://doi.org/10.1186/s12950-022-00313-y**

Following the publication of the original article [1], it was reported that errors occurred in Figs. 4 and 7. Regarding these concerns, the authors explained that the duplicated part of the image represents the same group of subjects (all belonging to the same index within the same processing group). The authors made a mistake when selecting panels for those figures. There was no significant effect on the results.

Below are the incorrect & updated figures:

**Incorrect**

Fig. 4


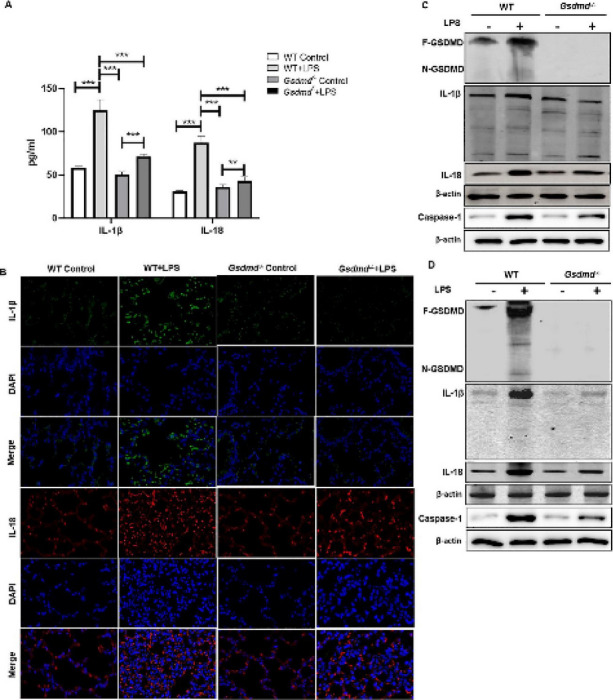


**Updated**

Fig. 4


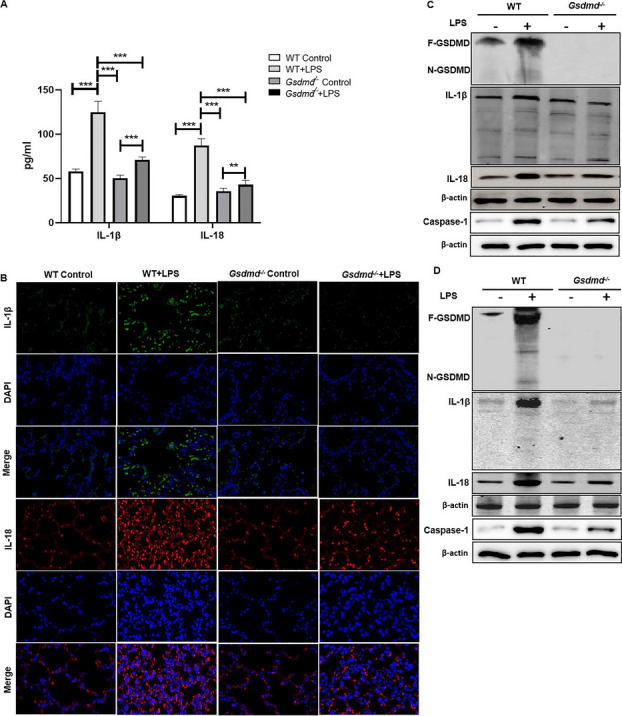


**Incorrect**

Figure 7


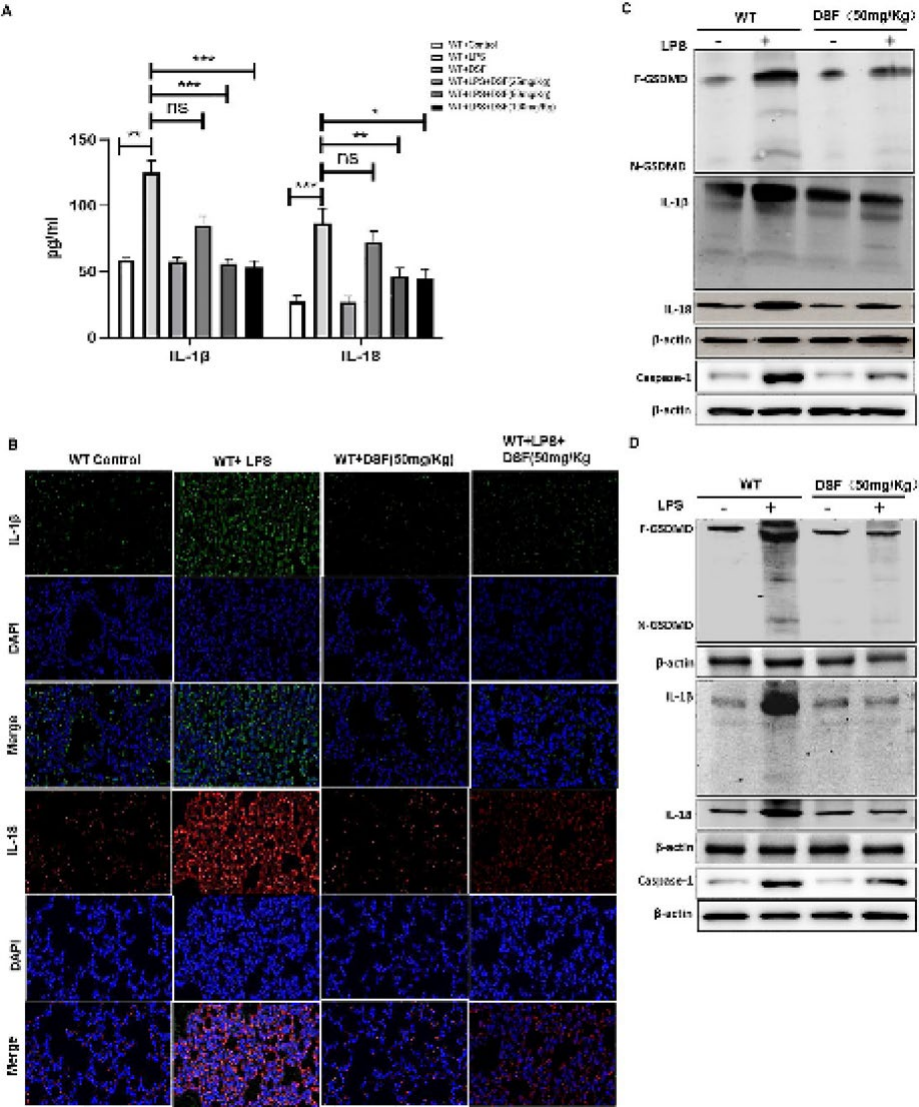


**Updated**

Figure 7


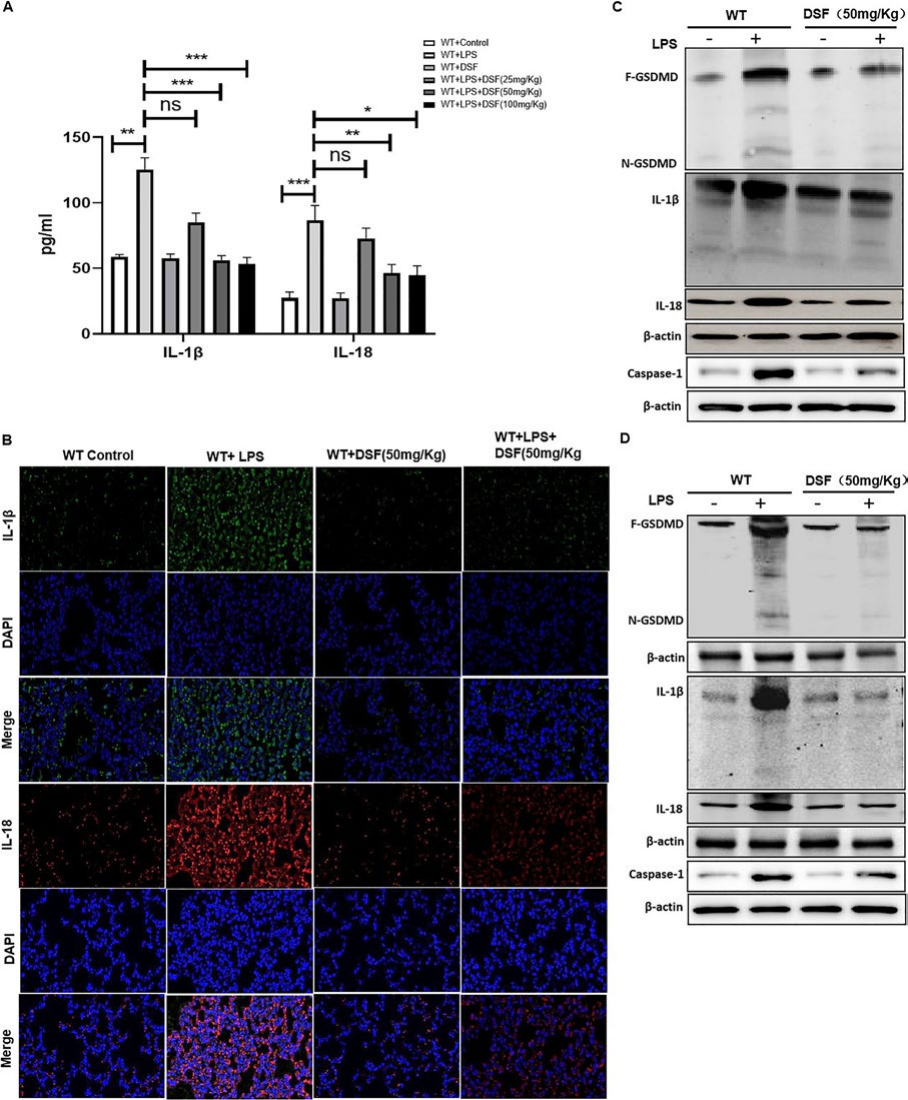


The Original Article has been corrected.
