# Supplementary material for: Disulfiram alleviates acute lung injury and related intestinal mucosal barrier impairment by targeting GSDMD-dependent pyroptosis
Source: J Inflamm (Lond). 2022 Oct 20;19:17. doi: 10.1186/s12950-022-00313-y (PMC9582395; doi:10.1186/s12950-022-00313-y)
Supplement: Supplementary file 1 — Supplementary Material 1 [file 12950_2022_313_MOESM1_ESM.docx]

**Supplemental Material**

**Disulfiram Alleviates Acute Lung Injury and Related Intestinal Mucosal Barrier**

**Impairment by Targeting GSDMD-Dependent Pyroptosis**

Jiping Zhao^1#^, Hong Wang^2#^, Jintao zhang ^3^, Fuwei Ou ^4^, Junfei Wang^1^, Tian Liu^1^, Jinxiang Wu^1*^

^1^ Department of Pulmonary and Critical Care Medicine, Qilu Hospital, Cheeloo College of Medicine, Shandong University, Jinan, China, ^2^ Department of Ophthalmology, Qilu Hospital, Cheeloo College of Medicine, Shandong University, Jinan, China, ^3^ Department of Respiratory, Shandong Qianfoshan Hospital, Cheeloo College of Medicine, Shandong University, Jinan, China ^4^ Yanzhou Branch of Affiliated Hospital of Jining Medical University, Jining, China,

**#** These authors have contributed equally to this work

*Correspondence: Jinxiang Wu, wdwujinxiang@126.com

**Supplementary Figure 4**

**Figure 4C**

**
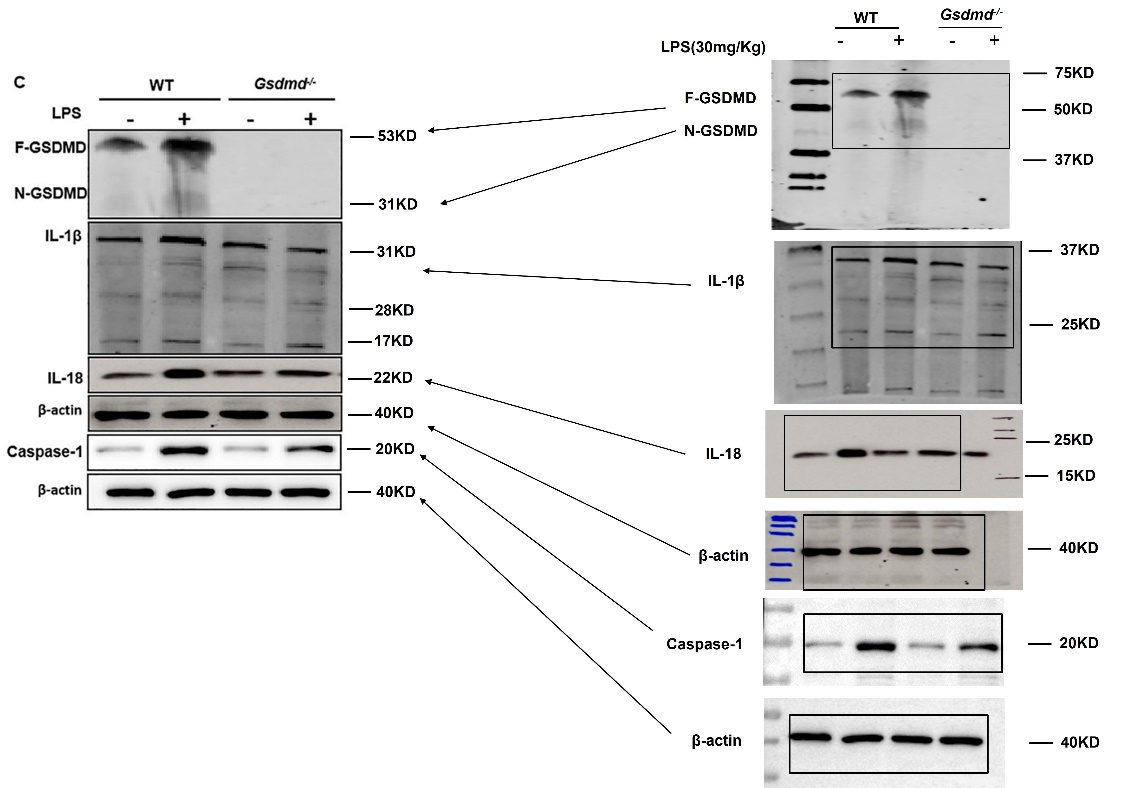
**

**Figure 4D**

**
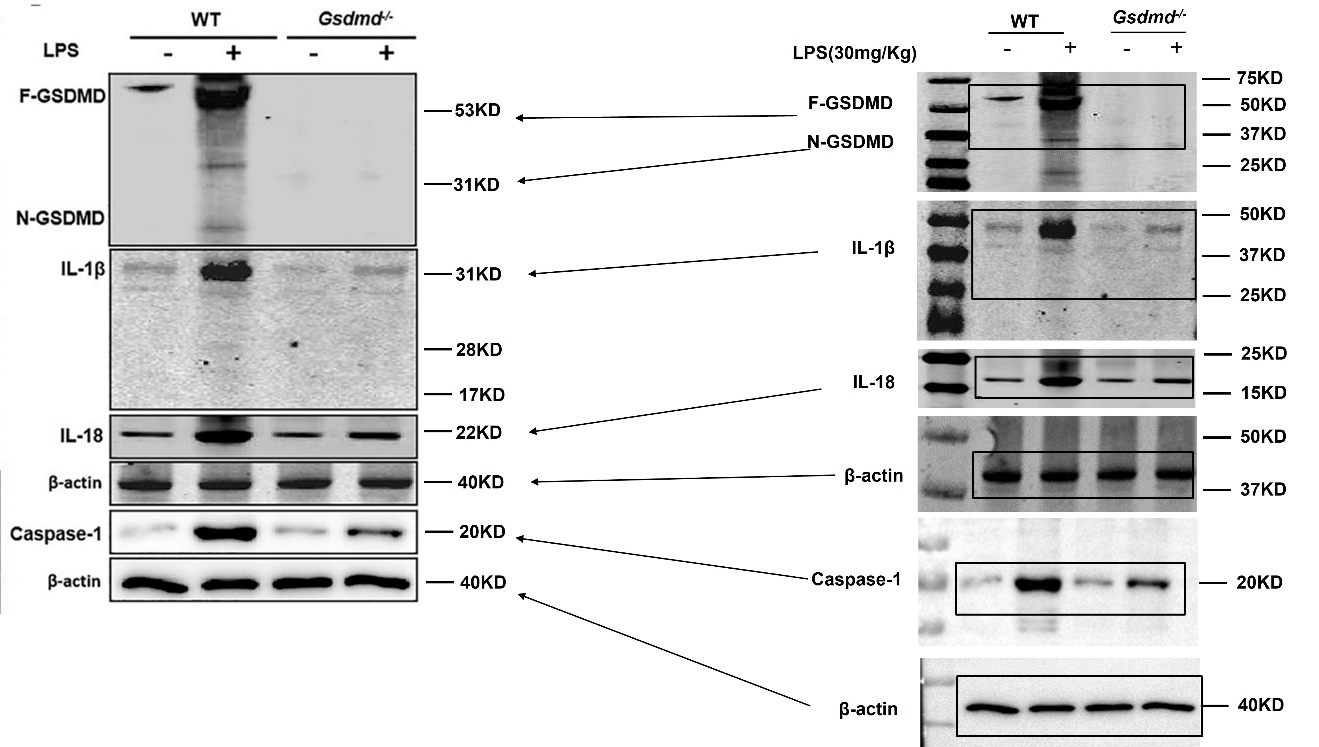
**

**Figure 7C**

**
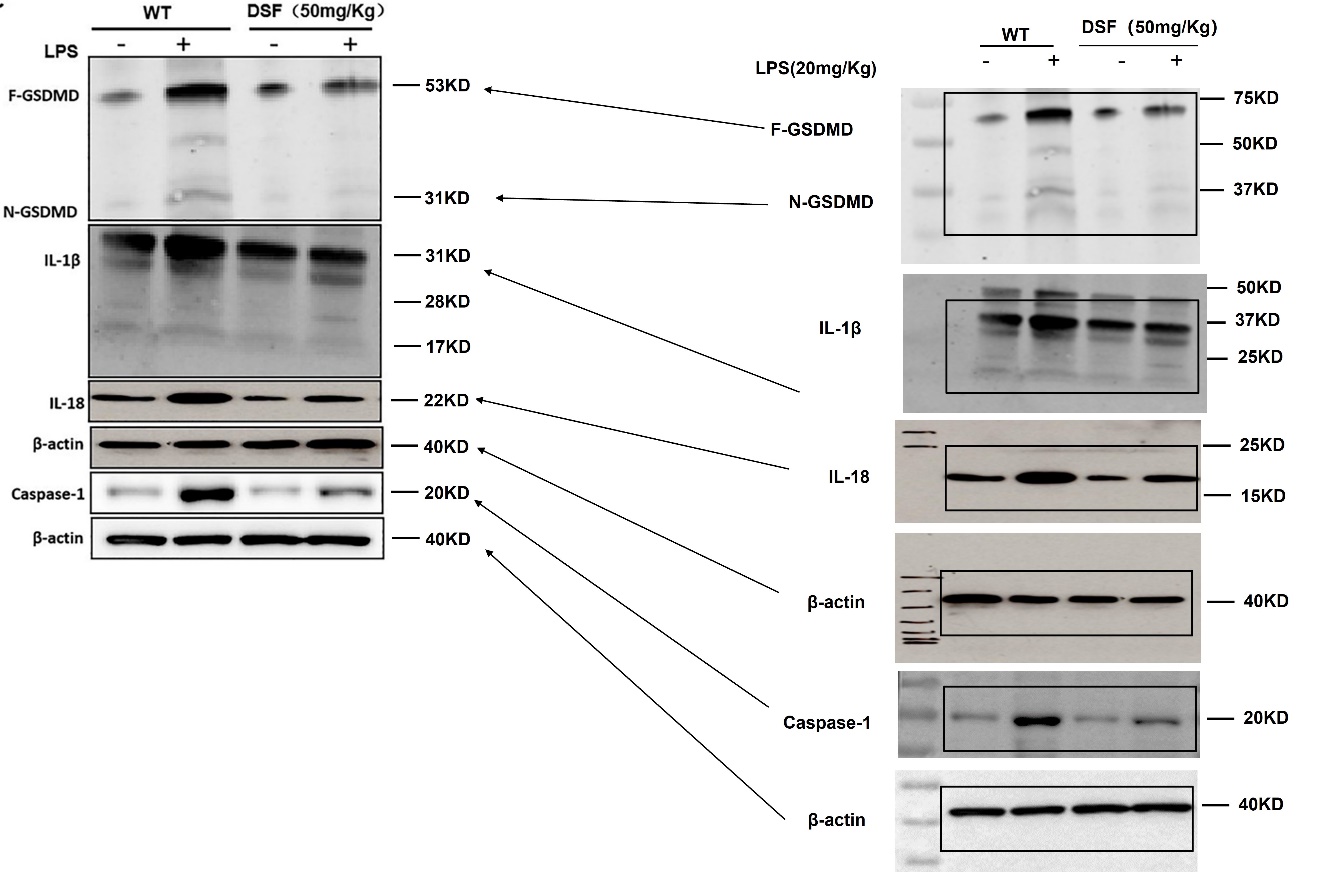
**

**Figure 7D**

**
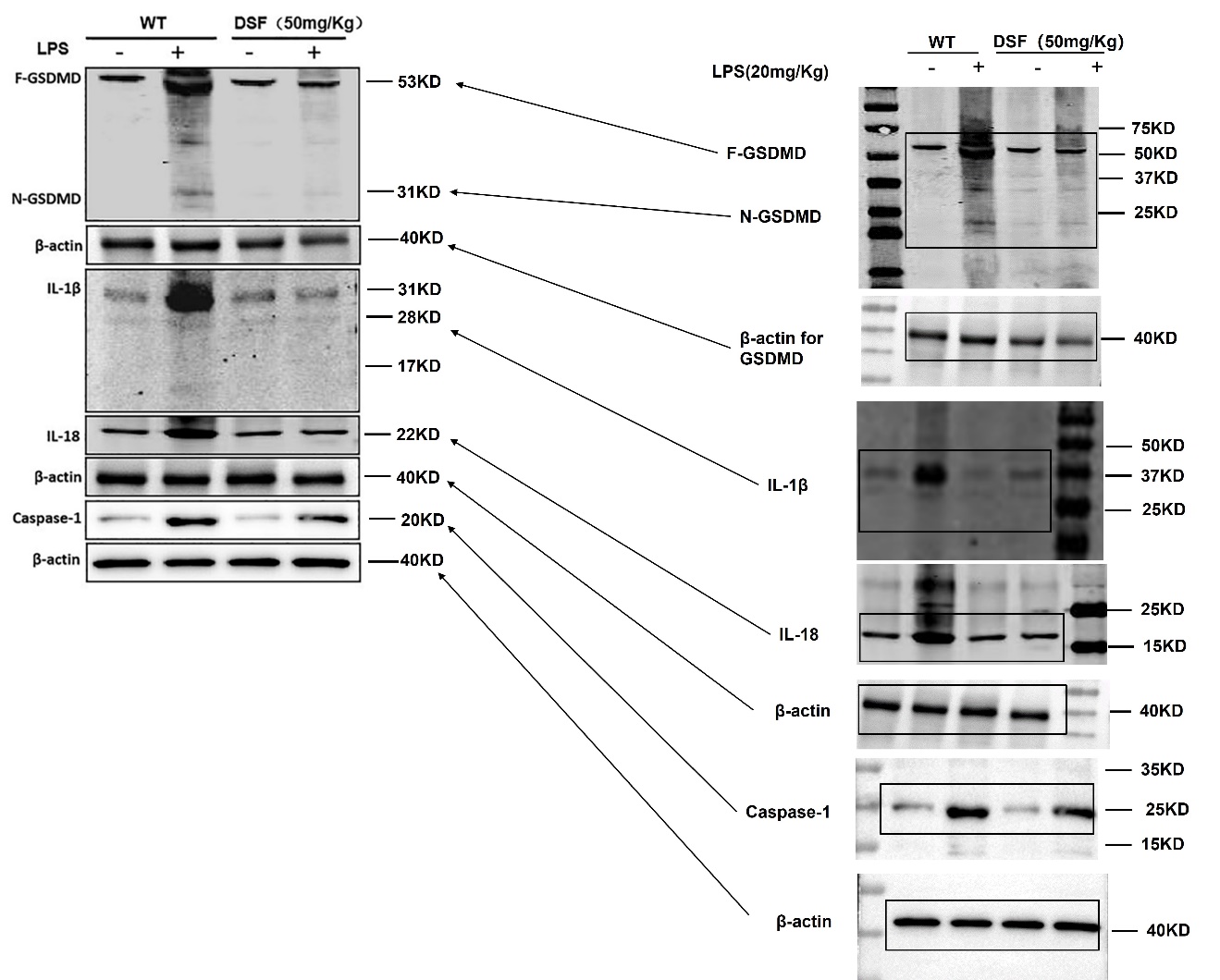
**
